# The Development and Evaluation of a Low-Cost, High-Fidelity Ultrasound-Guided Abdominal Paracentesis Simulator

**DOI:** 10.7759/cureus.93321

**Published:** 2025-09-27

**Authors:** Muhammed Shahid, Eren Keskin, Robert Pritchard, Azith Kalilul Rahman, Hajnalka Huszka, Habib Syed, Johann Willers, Alexander Hall

**Affiliations:** 1 Medicine, Brighton and Sussex Medical School, Brighton, GBR; 2 Anaesthetics, University Hospitals Sussex NHS Foundation Trust, Brighton, GBR; 3 Radiology, University Hospitals Sussex NHS Foundation Trust, Brighton, GBR

**Keywords:** abdominal paracentesis, ascites, confidence, high-fidelity, low-cost, simulation, ultrasound

## Abstract

Introduction

Paracentesis is a vital procedure for diagnosing and managing ascites, yet hands-on training opportunities are limited due to issues related to patient availability and procedural risks. Simulation-based training helps develop skills and confidence, but high-fidelity commercial models remain expensive and scarce. To address this gap, we created a novel, low-cost, high-fidelity ultrasound-guided abdominal paracentesis (USGAP) simulation model. Designed for affordability and realism, this model aims to improve trainee confidence and integrate ultrasound guidance to enhance procedural safety and precision.

Methods

A 3-litre saline bag was modified to simulate the abdominal cavity, incorporating aqueous dietary fibre antifreeze mix gel for liver tissue, fluid-filled sausage casing for the bowel, and dyed water for ascitic fluid. The model was sealed and coated with latex for self-sealing properties, with a total cost of £25. Assessment took place over two sessions (January-February 2025) involving 19 participants (senior house officers (SHOs), registrars, and consultants). Pre- and post-training questionnaires assessed training opportunities, confidence, and model effectiveness using a five-point Likert scale. Data was analysed using IBM SPSS Statistics (IBM Corp., Armonk, NY) (Wilcoxon signed-rank test, p<0.05). Ethics approval was not required as this study was registered as a quality improvement project. Consent was obtained, and all responses were anonymised.

Results

Pre-training data revealed significant gaps in USGAP training, with only 31.6% of participants reporting adequate exposure (median Likert 2/5). However, all participants (100%) recognised the importance of high-quality simulation. Following model use, confidence in performing USGAP significantly improved from 47.3% to 78.9% (p = 0.005). The model received unanimous positive feedback (100%) with participants praising its affordability, realism, and procedural accuracy.

Conclusions

This study demonstrated that a cost-effective, high-fidelity USGAP model significantly enhances trainees’ confidence while providing a sustainable and accessible alternative to expensive commercial models. The realistic anatomical structures, ultrasound capability, and self-sealing properties make it a valuable training tool. While the sample size was limited, the findings strongly support broader implementation into medical training. Future research should assess long-term skill retention and multi-centre adoption.

## Introduction

Peritoneal fluid is an essential physiological component of the peritoneal cavity that minimises friction between the visceral and parietal peritoneum. The peritoneal cavity normally contains approximately 20-50 mL of this fluid, maintained by a fine balance between the rate of fluid production and absorption [1]. Certain pathologies may disrupt this balance, leading to an accumulation of peritoneal fluid within the cavity, termed ‘ascites’. Causes of ascites can be classified into portal hypertension or non-portal hypertension, such as malignancy and infection. The most common cause of ascites is cirrhosis, which causes portal hypertension, followed by malignancy and heart failure [2,3]. 

Untreated ascites has numerous complications, including spontaneous bacterial peritonitis [4]. Paracentesis is a simple procedure that can be used to treat ascites, which involves inserting a needle into the peritoneal cavity [5]. Paracentesis can be diagnostic, whereby small volumes of ascitic fluid are removed for analysis to determine the causes of ascites, or therapeutic, in which large volumes of fluid are drained for symptomatic relief and to minimise the risk of complications occurring as mentioned earlier [6]. Serious complications of the procedure include fluid leak (3-13%), bleeding (0-2.7%), and organ puncture (<1%) [2,3,7]. Paracentesis is becoming an increasingly common procedure in clinical practice. Within the United States, Medicare-fee-for-service beneficiaries identified 150,000 paracenteses performed in 2008, a two-fold increase from 1993 [8]. Nationwide Inpatient Sample data saw a 10% increase in hospitalised patients with cirrhosis receiving paracentesis from 2004 (50%) to 2012 (61%, p<0.001) [9,10].

Given how common paracentesis is, physicians require good proficiency to perform the procedure safely and minimise the risk of complications. Moreover, within the UK, abdominal paracentesis is a competency sign-off that must be obtained by internal medicine trainees (IMTs), according to the Joint Royal College of Physicians Training Board (JRCPTB) [11]. However, numerous trainees report loss of training opportunities, insufficient time allocated for supervised practice, and difficulties in developing procedural skills competencies, with the majority attributing this to service provision [12,13]. Moreover, IMTs rotate across various specialties on a six-month basis. Consequently, the level of exposure to practical procedures, such as abdominal paracentesis, is partially dependent on the specific specialty in which a trainee is currently placed. This variation means some trainees gain greater exposure to these procedures compared to others. One study by Tasker et al. reported that among 871 IMTs, one in five had not performed therapeutic paracentesis, despite it being a competency sign-off by the JRCPTB [11,12].

Given that some trainees have limited opportunities to develop procedural competency, simulation can act as an effective tool to bridge the gap between infrequent procedural exposure and skill enhancement. Simulation has been shown to improve psychomotor procedural skills and confidence in low-risk, controlled environments [14-16]. Additionally, simulation provides trainees the opportunity to revisit and retain procedural competence months and years later [16]. Furthermore, JRCPTB informs IMTs that all practical procedures should be taught in simulation as early as possible [11].

Despite the growing benefit of simulation in clinical practice, commercial simulation models are costly and limited in availability. This study explores the development of a low-cost, high-fidelity ultrasound-guided abdominal paracentesis (USGAP) model. Ultrasound has been shown to significantly reduce complications of abdominal paracentesis, and evidence-based recommendations by the Society of Hospital Medicine recommend that ultrasound should be used to identify needle insertion sites [8]. In cases where fluid pockets are small or difficult to assess, real-time ultrasound should be used [17,18]. In this study, the model enabled trainees to use ultrasound to practice identifying suitable needle insertion sites. In some cases, real-time ultrasound was used to guide the needle. The primary aim of this study was to determine if a low-cost USGAP model can be used as an effective training tool to improve trainees’ confidence in performing abdominal paracentesis.

## Materials and methods

Development of the model

A 3-litre saline bag was used to replicate the abdominal cavity. A corner of the saline bag was cut off, and the saline was drained. The liver was made up of aqueous dietary fibre antifreeze mix gel (ADAMGel), a low-cost, high-fidelity tissue that closely resembles that of human anatomy [19]. The liver was covered with two layers of cling film to make it waterproof. Ultrasound gel was placed between the two layers of cling film to minimise air between the two layers. The removal of air from the model was an essential step in the model development, as ultrasound waves are not transmitted through air. The liver was then secured at the bottom of the bag using superglue. A sausage casing 80 cm in length was filled with fluid retention granules to replicate the bowel. The bag was then sealed with an iron and superglue. A giving set was inserted into the bag, and the bag was filled with water dyed yellow to represent ascitic fluid. Excess air was removed from the bag containing the ascitic fluid to create a vacuum. The bag was finally sprayed with contact adhesive and coated with three layers of liquid latex to achieve the model’s self-sealing properties. A layer of ADAMGel was placed on top to represent the abdominal muscles.

Assessment of the model

Assessment of the model took place over two sessions between January and February 2025, with both sessions facilitated by the same physicians who were competently trained in performing USGAP. The equipment used in both sessions included a Bonanno suprapubic catheter kit, identical to those used in clinical practice, a 10-ml syringe, an orange (25G) needle, and a Butterfly 2.0 ultrasound probe connected to an iPad installed with the compatible Butterfly iQ application. Teaching involved a live demonstration on the model, followed by questions from participants. Nineteen participants were involved in the study, including 10 senior house officers (SHOs), seven registrars, and two consultants.

Facilitators were present throughout the training session to support participants and provide direct feedback on how to optimise their technique in performing the procedure. Participants completed a questionnaire that assessed both pre- and post-model use. Before using the model, questions focused on confidence in performing USGAP, opportunities to practice the procedure, views on the importance of simulation, trainee access to simulation, and whether NHS trusts would benefit from the simulation model. After using the model, the same questions were asked, along with additional questions about the model's use in training and simulation. Questions were answered in the form of a modified five-point Likert scale from a previous study (1/5 = Strongly Disagree, 2/5 = Disagree, 3/5 = Neutral, 4/5 = Agree, 5/5 = Strongly Agree) [20].

Analysis

Statistical analysis of data was conducted using IBM SPSS Statistics (IBM Corp., Armonk, NY) for Windows [21]. Given that the data were ordinal and did not follow a normal distribution, the Wilcoxon signed-rank test was used to determine the medians, interquartile ranges (IQR), p-values (<0.05), and 95% confidence intervals (CI). 

Ethics 

This study was registered by the University Hospital Sussex NHS Foundation Trust as a quality improvement project by the clinical governance team. Therefore, formal ethics approval by the research ethics committee was not required. Consent was obtained from all physicians involved in the study. All questionnaire responses were anonymous.

## Results

Nineteen clinicians participated in the study, with data collected before and after model use. Data were then analysed. Pre-questionnaire responses assessing the degree of opportunities available to perform ultrasound-guided abdominal paracentesis, and the importance and the demand for simulation models are presented in Table 1.

**Table 1:** Pre-questionnaire responses assessing degree of opportunities available to perform ultrasound-guided abdominal paracentesis, and the importance and demand for simulation models

Statement	% Agree/strongly agree (n)	Median	Interquartile range	Range
I have been provided ample training opportunities in clinical practice to learn and maintain the skills related to ultrasound-guided abdominal paracentesis	31.6% (6)	2	3	1-5
Simulated training sessions are important for improving trainees’ confidence in performing ultrasound-guided abdominal paracentesis	100% (19)	5	0	4-5
NHS Foundation Trusts would benefit from having ultrasound-guided abdominal paracentesis training models	100% (19)	5	0	4-5

Post-questionnaire results showed the changes in trainees’ confidence following model use and statements regarding the model’s effectiveness as a training tool for trainees. These results are shown in Tables 2-3.

**Table 2:** Data assessing confidence in performing USGAP before and after model use Statistical analysis was conducted using the Wilcoxon signed-rank test USGAP: ultrasound-guided abdominal paracentesis

Statement	Before model use				After model use				p-Value	95% confidence interval
	% Agree/strongly agree (n)	Median	Interquartile range	Range	% Agree/strongly agree (n)	Median	Interquartile range	Range		
I feel confident in performing ultrasound-guided abdominal paracentesis	47.3% (9)	3	2	1-5	78.9% (9)	4	1	2-5	0.005	0.43-1.78

**Table 3:** Post-questionnaire data assessing clinicians’ perspectives on the quality of the model as a training tool (agree = Likert 4/5 and strongly agree = Likert 5/5)

Statement	% Agree/strongly agree (n)	Median	Interquartile range	Range
The model reasonably resembles the real thing	84.2% (16)	4	0	2-5
The model is a good training tool for inexperienced trainees	100% (19)	5	0	4-5
Trainees should practice with this model before attempting the procedure on patients	100% (19)	5	1	4-5
I would use this model to teach ultrasound-guided abdominal paracentesis	100% (19)	5	1	4-5

Results from the pre-questionnaire, assessing the extent of training opportunities, the importance of simulation, and the demand for USGAP simulation models, are shown in Table 1. Results showed that only 31.6% of participants agreed or strongly agreed that they had access to training opportunities to learn or maintain the USGAP-related skill, with a median Likert score of 2/5 across all participants' responses. These results demonstrate the lack of training opportunities currently available. Uniformly, all participants agreed or strongly agreed that simulation was important to learn or maintain the skill USGAP and that NHS Trusts would benefit from having access to the USGAP model. In both cases, results showed a median Likert score of 5/5. These results confirmed the need for the course, the model, and ultimately this study.

**Figure 1:**
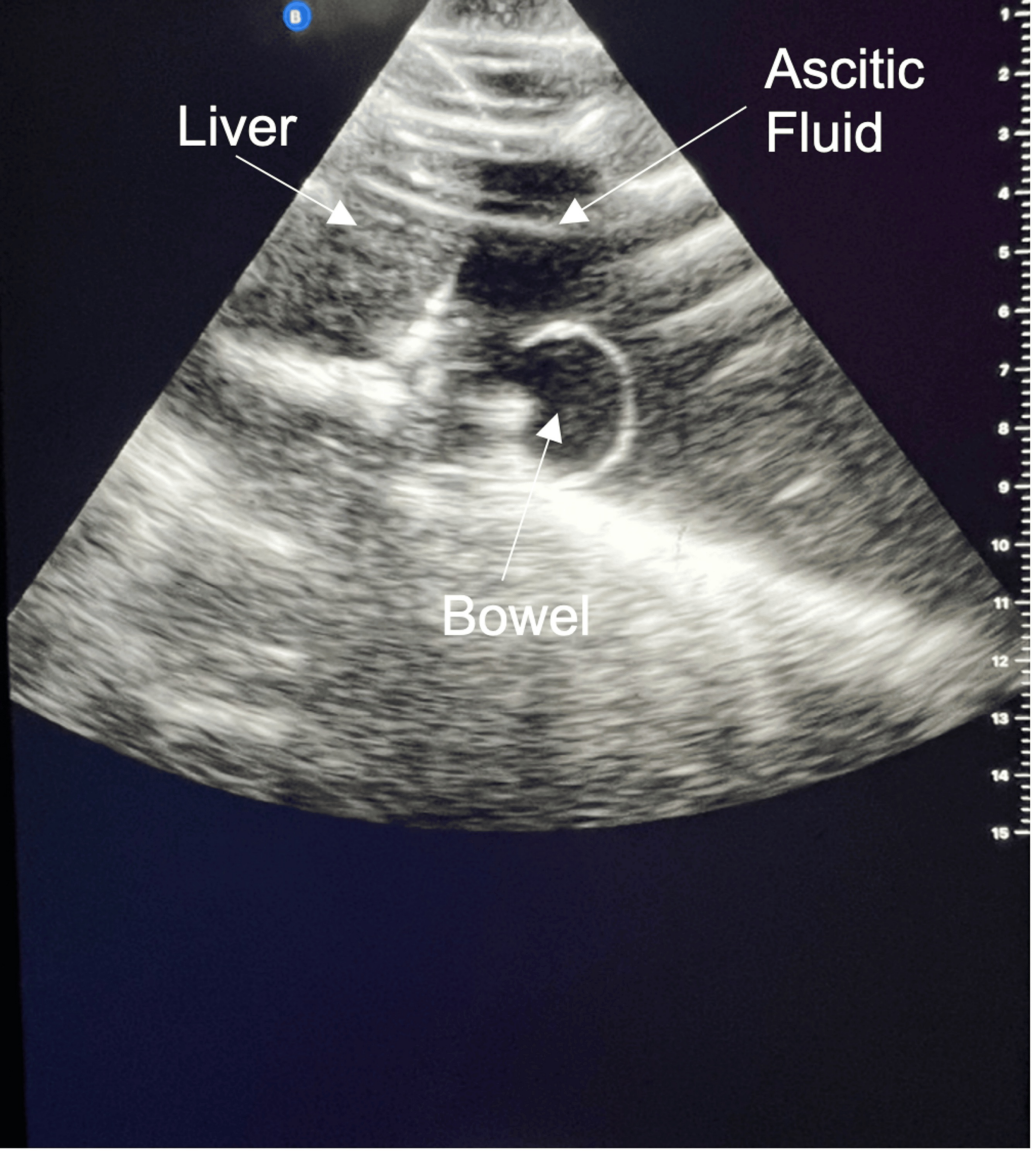
USGAP model under ultrasound showing presence of liver, bowel and ascitic fluid labelled Depth is shown on the right-hand side in centimetres USGAP: ultrasound-guided abdominal paracentesis

**Figure 2:**
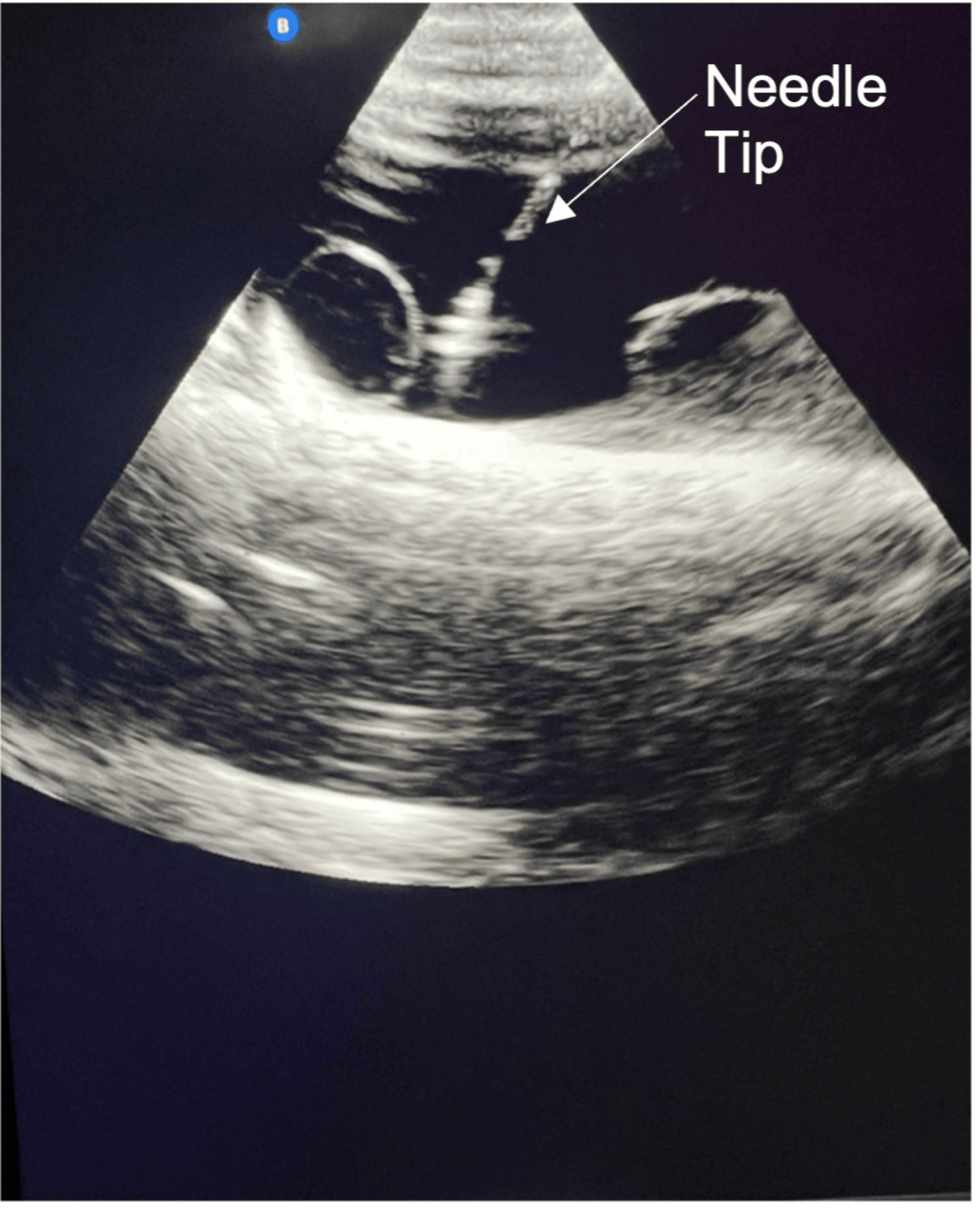
USGAP model with suprapubic catheter being guided into the ascitic fluid pocked using real time ultrasound USGAP: ultrasound-guided abdominal paracentesis

Data collected following use of the model (Table 2) demonstrated a statistically significant improvement in trainees’ confidence in performing USGAP with 47.3% of participants agreeing or strongly agreeing to be confident at performing the procedure before model use and 78.9% agreeing or strongly agreeing to be confident at performing the procedure after model use (p = 0.005, 95% CI 0.43 - 1.78). The median Likert scale score also increased from 3 to 4 following model use. Figures 1-2 show the model under ultrasound, with Figure 2 also showing needle guidance under ultrasound.

Additional post-questionnaire data in Table 3 showed that 84.2% of participants agreed or strongly agreed that the model reasonably resembled the real thing, with a median Likert score of 4/5. Uniformly, all participants agreed or strongly agreed that the model is a good training tool for inexperienced trainees, that trainees should practice with the model before attempting the procedure on patients, and that they would use the model as a teaching tool, with a median Likert score of 5/5. 

Qualitative data included descriptive feedback by clinicians. Comments included ‘good replica of real-life anatomy’, ‘very realistic’, ‘ability to test without having to do this on patients’, ‘boosts confidence for practising ascitic drains’, and ‘good practice simulation model to help build confidence in radiology trainees on performing ultrasound-guided procedures. These comments demonstrate the potential effectiveness of using the USGAP model as a training tool in future practice to increase clinicians’ proficiency in performing the procedure.

## Discussion

Several studies have reported Insufficient opportunities to perform USGAP [12,13]. This is largely problematic given how common USGAP is and therefore requires clinicians to have good proficiency in it. Current commercial simulation models available are expensive and thus not easily accessible for training [16]. This study aimed to provide a solution to this by designing a low-cost, high-fidelity USGAP model. Results have demonstrated both the demand for the model and its potential effectiveness as a training tool. The model has been shown to improve trainees’ confidence and has been rated highly in multiple domains, assessing its quality and effectiveness as a training tool.

The model possesses several advantages compared to commercial models. Most crucially, the cost to manufacture the model is significantly less, at approximately £25.00. This is significantly less than the cost of commercial models, which can cost up to £13,000 [22]. The inexpensive price to manufacture the model is key to improving accessibility to these simulation models and ultimately improving physicians’ clinical proficiency in performing USGAP. Moreover, multiple models can be more easily manufactured at a lower cost, thus increasing accessibility to NHS Trusts around the UK. Furthermore, our models feature additional visceral organs, such as the liver, that look realistic under ultrasound with mobile bowel. This is evident from comments left by physicians such as ‘good replica of real-life anatomy’.

Other important features include our model’s ability to be punctured more than 40 times before any significant leaks begin occurring. Nevertheless, leaks can be easily patched with super glue, and latex can be reapplied to create a new seal. The ability to withstand multiple punctures is crucial for enhancing physicians' proficiency. A review by Elendu et al. [23] on simulation in medical education highlights the benefits of deliberate practice, allowing trainees to perform procedures repeatedly in a safe environment. This repetition improves skill acquisition, retention, and confidence while reducing anxiety [24-25].

The model can be customised by varying the amount of bowel and ascitic fluid during both the manufacturing process and teaching sessions. This allows the difficulty level to be adjusted according to the physician's skill, making it suitable for trainees at different stages of proficiency. When performing abdominal paracentesis on the simulation model, ultrasound can be used to help locate suitable ascitic pockets for drainage or provide real-time needle guidance, especially when the pockets are small. This is made possible by the ability to adjust the fluid levels during the teaching sessions. Thus, the model can be tailored to different proficiency levels and different specialities, such as radiology, where the trainees typically perform abdominal paracentesis under real-time ultrasound. 

To ensure the relevance of the study, several similar studies were identified in the literature [26-29]. A 2024 study by Huang et al. [26] created an agar-based ultrasound-guided paracentesis phantom. The design of this model involves a balloon used to represent the peritoneal cavity, a cotton rope to represent the bowel, and agar to represent the human skin and subcutaneous area. The course included formal training, with all trainees showing improved confidence in performing USGAP following model use, compared with pre-training when assessed using a 5-point Likert scale (4 vs. 1, p<0.001). The model was low-cost, at approximately $16, and emphasised the potential of using low-cost simulation models to improve accessibility and clinical proficiency in performing such procedures. Our study further reinforces the growing evidence on the use of low-cost simulation models in clinical training.

A Brazilian study conducted by Silveira et al. [27] explored a different model style using a plastic bottle filled with yellow gouache paint and covered with silicon and D28 foam to represent the peritoneal cavity, muscle layers, and skin. This was placed within the left iliac fossa of a cut-out mannequin. A training course involving 51 medical students showed an 82.3% improvement in confidence (p<0.0001) regarding performing abdominal paracentesis following training on the model. This simulation model costs approximately £13. Although participants in the study could improve their manual dexterity for needling, they did not use ultrasound, unlike in our study. This meant that enhancing anatomical familiarity and needle guidance was not possible, which are key to boosting confidence and safely performing abdominal paracentesis on patients.

Other similar studies included a 2018 Brazilian study involving a manikin filled with multiple gloves, each containing different coloured fluids to represent blood and ascitic fluid, with one glove filled with enteric content [28]. Further development of the model resulted in a model that could be punctured 30 times for $22 (£16.23). Of 87 participants, 97.7% agreed that the model is easily reproducible, and all participants agreed that the model should be used before performing the procedure on patients.

A study in Chile used a 3D-printed abdominal paracentesis simulation model to assess 69 fourth-year medical students. The study measured students' ability to perform therapeutic paracentesis, with results indicating significant improvement in procedural skills (pre-evaluation: 13.36 ± 4.46 vs. post-evaluation: 22.3 ± 1.83 points, p<0.01). These findings align with other studies in the literature, highlighting the effectiveness of low-cost simulation models in enhancing training and procedural proficiency. However, the study did not incorporate ultrasound, and as it involved medical students, its results may have limited generalisability due to the students' lower baseline clinical experience and anatomical familiarity compared to practising physicians.

Limitations and future directions

This study has several limitations. First, the small sample size and single-centre design precluded sample size calculations, limiting the generalisability of the results. Expanding the study to multiple centres and incorporating more ultrasound-based teaching sessions could address this limitation. Additionally, participant awareness that their responses may be used in future studies could have introduced response bias. There was also variability in participants' confidence levels, with senior physicians (registrars and consultants) displaying higher confidence in performing USGAP on the simulation model compared to junior physicians (e.g., SHOs). This disparity may affect the generalisability of the findings across different experience levels. However, the simulation model primarily benefits junior physicians, who generally have less experience with abdominal paracentesis. Notwithstanding this, the adjustable fluid pocket size in the model allows for tailored difficulty levels, making it suitable for both junior and senior physicians. Moreover, smaller fluid pocket sizes facilitate real-time ultrasound use, enabling radiology trainees to also benefit from the model.

The model’s physical design was limited to a 3-litre capacity, restricting its ability to simulate anatomically complex patients, such as those with a higher BMI or large-volume ascites (≥5L). Additionally, the study did not formally assess procedural competence through a checklist or structured evaluation, relying instead on participants' self-reported confidence after practising the procedure. While this approach aligned with the study’s aim of enhancing confidence rather than replacing patient-based practice, it limits conclusions about actual skill acquisition. Nonetheless, the model provided a safe environment for building procedural confidence, a critical step toward clinical competence.

Another limitation was the lack of long-term follow-up to assess whether increased confidence translated into improved performance on real patients. Future sessions could benefit from including education on the indications and contraindications of USGAP. Furthermore, future research should incorporate formal assessments of procedural confidence, skill retention, and the ability to successfully perform the procedure on real patients post-simulation.

## Conclusions

Ultrasound-based simulation is a growing area within clinical medicine, with a limited number of low-cost, high-fidelity simulation models currently available. This study successfully demonstrated the effectiveness of using an adaptable ultrasound-based abdominal paracentesis model to improve physicians’ confidence and proficiency in performing USGAP. Results showed clear improvements in physicians’ clinical confidence in performing the procedure, with all physicians favouring using the model as a training tool. Ultrasound-based simulation is limited in many areas globally due to the high costs of obtaining simulation models. Formal assessment of procedural skill and competency was not studied; however, this could be an area of future research. This study aims to provide a low-cost alternative that can improve access to ultrasound-based simulation training.

## Appendices

Ultrasound-guided abdominal paracentesis questionnaire feedback

When you submit this form, it will not automatically collect your details, like name and email address, unless you provide them yourself.

Required

1. Deanery/Region

2. Grade

**Table 4:** Question 2. Grade

Grade
FY1/FY2
Core Trainee or Clinical Fellow
Registrar
Consultant
Other

3. Please answer the following statements before using the model

**Table 5:** Question 3. Please answer the following statements before using the model

	Strongly Disagree	Disagree	Neutral	Agree	Strongly Agree
I feel confident in performing ultrasound-guided abdominal paracentesis					
I have been provided ample training opportunities in clinical practice to learn and maintain the skill of ultrasound-guided abdominal paracentesis					
Simulated training sessions are important for improving trainees’ confidence in performing ultrasound-guided abdominal paracentesis					
NHS Foundation Trusts would benefit from having ultrasound-guided abdominal paracentesis training models					

4. Please answer the following questions after the model use.

**Table 6:** Question 4. Please answer the following statements after model use

	Strongly Disagree	Disagree	Neutral	Agree	Strongly Agree
I feel confident in performing ultrasound-guided abdominal paracentesis					
The model reasonably resembles the real thing					
The model is a good training tool for inexperienced trainees					
Trainees should practice with this model before attempting the procedure on patients					
I would use this model to teach ultrasound-guided abdominal paracentesis					

Question 5. Any comments on what you found good or bad?
